# *Candida albicans* exposures, sex specificity and cognitive deficits in schizophrenia and bipolar disorder

**DOI:** 10.1038/npjschz.2016.18

**Published:** 2016-05-04

**Authors:** Emily G Severance, Kristin L Gressitt, Catherine R Stallings, Emily Katsafanas, Lucy A Schweinfurth, Christina L Savage, Maria B Adamos, Kevin M Sweeney, Andrea E Origoni, Sunil Khushalani, F Markus Leweke, Faith B Dickerson, Robert H Yolken

**Affiliations:** 1 Stanley Division of Developmental Neurovirology, Department of Pediatrics, Johns Hopkins University School of Medicine, Baltimore, MD, USA; 2 Sheppard Pratt Health System, Stanley Research Program, Baltimore, MD, USA; 3 Department of Psychiatry and Psychotherapy, Central Institute of Mental Health, Medical Faculty Mannheim, Heidelberg University, Mannheim, Germany

## Abstract

Immune aberrations in schizophrenia and bipolar disorder have led to the hypotheses that infectious agents or corresponding immune responses might contribute to psychiatric etiopathogeneses. We investigated case–control differences in exposure to the opportunistic fungal pathogen, *Candida albicans,* and examined associations with cognition, medication, lifestyle, and somatic conditions. We quantified *C. albicans* IgG antibodies in two cohorts totaling 947 individuals and evaluated odds ratios (OR) of exposure with psychiatric disorder using multivariate regressions. The case–control cohort included 261 with schizophrenia, 270 with bipolar disorder, and 277 non-psychiatric controls; the second included 139 with first-episode schizophrenia, 78 of whom were antipsychotic naive. No differences in *C. albicans* exposures were found until diagnostic groups were stratified by sex. In males, *C. albicans* seropositivity conferred increased odds for a schizophrenia diagnosis (OR 2.04–9.53, *P*⩽0.0001). In females, *C. albicans* seropositivity conferred increased odds for lower cognitive scores on Repeatable Battery for the Assessment of Neuropsychological Status (RBANS) in schizophrenia (OR 1.12, *P*⩽0.004), with significant decreases on memory modules for both disorders (*P*⩽0.0007–0.03). *C. albicans* IgG levels were not impacted by antipsychotic medications. Gastrointestinal (GI) disturbances were associated with elevated *C. albicans* in males with schizophrenia and females with bipolar disorder (*P*⩽0.009–0.02). *C. albicans* exposure was associated with homelessness in bipolar males (*P*⩽0.0015). In conclusion, sex-specific *C. albicans* immune responses were evident in psychiatric disorder subsets. Inquiry regarding *C. albicans* infection or symptoms may expedite amelioration of this treatable comorbid condition. Yeast exposure as a risk factor for schizophrenia and its associated cognitive and GI effects require further investigation including the possible contribution of gut–brain mechanisms.

## Introduction

Psychiatric disorders may arise from a combination of genetic and environmental factors, and increasingly, immune-related hypotheses are proposed to reconcile both aspects of this interface.^1–3^ One theme of psychiatric immune research has been the search for infectious, possibly neurotropic, pathogens as etiological agents.^4,5^ It is believed that exposure to infectious organisms such as viruses or parasites during critical stages of neurodevelopment may confer damage to the central nervous system, thus resulting in the onset of behavioral anomalies and psychiatric disorders during adulthood.^6,7^

Fungal pathogens have not been extensively evaluated in studies of psychiatric disorders. We have previously shown that people with schizophrenia and bipolar disorder have elevated antibodies to *Saccharomyces cerevisiae*,^8,9^ a yeast species commonly used by bakers and brewers and not typically considered to be virulent in humans. Studies of the human mycobiome regularly report the presence of *S. cerevisiae*, but *Candida albicans* and other Candidal species are the clearly dominant commensal fungus in humans.^10,11^ *C. albicans* is a diploid, polymorphic yeast residing in mucosal surfaces of the human respiratory, gastrointestinal (GI) and genitourinary tracts. Under a set of predisposing conditions, *C. albicans* can become pathogenic ranging in seriousness from local infections of the mouth, throat, and reproductive tract to a systemic invasive candidiasis that impacts the circulatory system, bones and brain.^12^ Although bacterial dysbioses can contribute to *C. albicans* overgrowth by failing to provide the competition needed to keep the fungus in check, it is likely that the complexities of the fungal–bacterial, fungal–fungal, and fungal–host relationships are currently underappreciated.^13^

Here we investigated *C. albicans* as a new candidate infectious disease target for studies of schizophrenia and bipolar disorder. Blood levels of IgG class antibodies directed against a specific pathogen generally reflect the lifetime exposure of an individual to that antigen.^14^ Thus, we measured and compared IgG antibodies directed against this fungus in two psychiatric cohorts: one composed of 261 people with schizophrenia, 270 with bipolar disorder and 277 individuals without a history of psychiatric disorder; the other cohort was composed of 139 people with first-episode schizophrenia, 78 of whom were antipsychotic naive. We then determined whether exposure to *C. albicans* was associated with cognitive symptoms, as evident by scores on the Repeatable Battery for the Assessment of Neuropsychological Status (RBANS) Form A.^15^ Finally, to provide a preliminary indication of whether or not *C. albicans* exposure could be considered a candidate risk factor for these psychiatric disorders, we investigated significant disease associations with outside, potentially confounding, influences. Toward this end, we evaluated our antibody measures in conjunction with a variety of extrinsic factors including antipsychotic medication, homelessness, and somatic comorbidities. Among the somatic conditions tested were cardiovascular, endocrine, GI, genitourinary, hepatic, neoplastic and respiratory disturbances.

## Results

**Figure 1:**
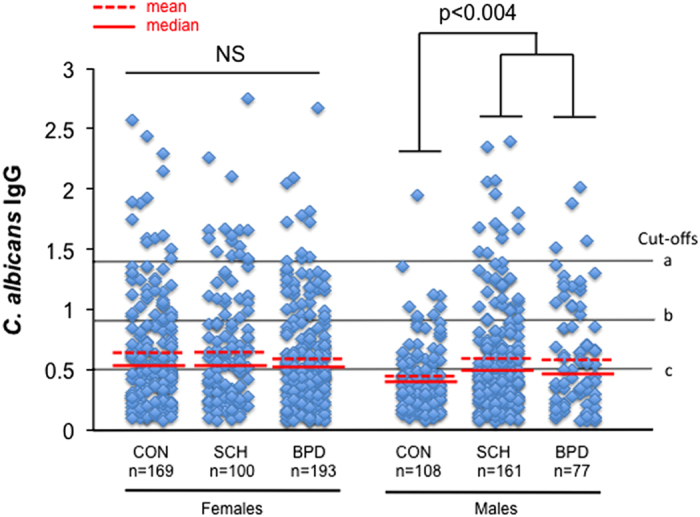
*C. albicans* IgG levels and seropositivity in schizophrenia and bipolar disorder compared to controls. Shown are data distributions for each diagnostic category according to sex. The letters, a-c, refer to seropositivity designations based on different calculated cutoff values (see Materials and Methods section). CON, non-psychiatric control; SCH, schizophrenia; BPD, bipolar disorder. Significant differences among means of the three male groups, but not female groups, were observed (ANOVA, *P*⩽0.0043, see Results section). Odds ratios are listed in Table 1.

First, we examined *C. albicans* IgG levels in cases compared with controls and found no significant differences among the diagnostic groups (analysis of variance (ANOVA) F=0.93, *P*⩽0.39). However, when we categorized groups according to sex, we found significant elevations of *C. albicans* IgG in males with schizophrenia and bipolar disorder compared with male controls (ANOVA F=5.55, *P*⩽0.0043; data distributions, means, and medians are shown in Figure 1). In multinomial logistic regression models that included the independent variables of age, homelessness, race, and socioeconomic status, we found that irrespective of the positivity cutoff designation used as the dependent variable, elevated *C. albicans* measures conferred increased odds ratios (OR) to males with schizophrenia compared with control males (Table 1). The elevated *C. albicans* IgG levels that we identified in bipolar disorder males were found to be a function of a positive homelessness history (positive, *n*=12, mean±s.d., 0.98±0.55; negative, *n*=65, mean±s.d., 0.53±0.40; *t*-tests, *t*=3.30, two-tailed *P*⩽0.0015). *C. albicans* antibody levels in females were not distinguishable between groups, but notably were of the same magnitude as the male schizophrenia and male bipolar disorder groups (Figure 1).

We measured *C. albicans* antibody levels in a second cohort of 139 individuals with first-episode schizophrenia, 78 of whom were antipsychotic naive. We detected no differences in antibody levels between those who were antipsychotic naive compared with those who were receiving antipsychotic medications (AP+, mean±s.d. 0.60±0.39; AP−, mean±s.d. 0.56±0.33; *t*-tests, *t*=0.72, two-tail *P*⩽0.47). There were also no differences between males in these two medication groups or between females in these two groups (AP+ male, *n*=30, mean±s.d. 0.52±0.29; AP− male, *n*=44, mean±s.d. 0.51±0.34; *t*-tests *t*=0.07, two-tail *P*⩽0.94; AP+ female, *n*=31, mean±s.d. 0.68±0.46; AP− female, *n*=34, mean±s.d. 0.61±0.31; *t*-tests, *t*=0.70, two-tail *P*⩽0.49). There were trends towards increased antibody levels in females compared to males in both medication groups (*t*-tests, *t*=1.36–1.62, *P*⩽0.06–0.08).

We then tested whether *C. albicans* positivity was associated with cognitive impairment. We found that females with schizophrenia who were *C. albicans* IgG-seropositive performed more poorly on these tests than did females with schizophrenia who were *C. albicans* IgG-seronegative or than female controls (Total RBANS score: multinomial logistic regression, OR 1.12, 95th% CI 1.03–1.23, *P*⩽0.004). Furthermore, performance on the Immediate and Delayed Memory modules of the RBANS test were significantly reduced in both *C. albicans* IgG-seropositive women with schizophrenia and those with bipolar disorder compared with respective seronegative groups, with some variation depending on the positivity model (Table 2). Male schizophrenia, male bipolar disorder, male control, and female control groups did not show significant differences in scores on the RBANS test that could be attributable to *C. albicans* IgG levels.

To determine whether a particular somatic or clinical condition affected the observed patterns of *C. albicans* antibodies, we compared IgG levels between individuals with psychiatric disorders who were positive and negative for current or chronic conditions in seven categories: cardiovascular, endocrine, genitourinary, GI, hepatological, neoplastic, and respiratory disturbances. Of the categories evaluated, GI, genitourinary and neoplastic conditions were associated with *C. albicans* levels, again in a sex-specific manner (Table 3). Reported GI symptoms included constipation, Crohn’s disease, diarrhea, gastroesophageal reflux disease, irritable bowel syndrome, lactose intolerance, Norwalk virus, pancreatitis, ulcers, any abdominal surgery, and any abdominal pain. Genitourinary disturbances included urinary tract infections, kidney infections, cysts, urinary incontinence, and sexually transmitted diseases. Neoplastic conditions included pituitary adenoma, melanoma, lung cancer, breast cancer, testicular cancer, endometrial cancer, stomach cancer, liver cancer and ovarian cancer. Significant *C. albicans*-associated GI symptomatology and genitourinary disturbances affected males with schizophrenia and females with bipolar disorder, although the genitourinary disturbances did not survive Bonferroni multiple comparison corrections. We also observed a trend toward association of *C. albicans* IgG with neoplastic conditions in males with schizophrenia (Table 3). To determine whether GI disturbances were significantly associated with antibody levels independently of a cancer history or genitourinary source, we reanalyzed the GI association with neoplasm- and genitourinary-positive individuals removed. The positive association of GI symptoms and elevated *C. albicans* IgG in both groups persisted, although these analyses did not survive Bonferroni multiple comparison corrections (males with schizophrenia, GI negative, *n*=67, mean±s.d. 0.50±0.36; GI positive, *n*=52, mean±s.d. 0.64±0.46; *t*-test, *t*=−1.83, one-tail *P*⩽0.03; females with bipolar disorder, GI negative, *n*=45, mean±s.d. 0.46±0.35; GI positive, *n*=42, mean±s.d. 0.64±0.52, *t*-test, *t*=−1.74, one-tail *P*⩽0.03).

## Discussion

Our results document that antibodies directed against the opportunistic fungal pathogen, *C. albicans*, were elevated in distinct subsets of individuals with psychiatric disorders. For some of these individuals, females in particular, *C. albicans* antibodies were associated with reduced cognitive functioning. Elevated *C. albicans* could be attributable to extraneous lifestyle or somatic variables for some subgroups, but for others, such as males with schizophrenia, no confounders were evident suggesting the possibility that exposure to this pathogen could be a risk factor for this psychiatric disorder. Sex-specific patterns reflected expectations that yeast overgrowth is found in the female reproductive tract, during immunosuppression and in association with homelessness. *C. albicans* antibody patterns in both sexes preliminarily implicated the GI tract as a source of microbial dysbioses in both schizophrenia and bipolar disorder. Follow-up studies are required to determine how unregulated *C. albicans* growth might impact the gut–brain axis in psychiatric disorders.

Many of our observations mirrored epidemiological and sex-specific expectations of heightened exposure to *C. albicans* in women, in those who were immunocompromised and in people who had a history of homelessness. In this regard, these antibody associations with lifestyle and somatic conditions represented conceptual controls. The portion of our analyses with males showed the most direct and strongest linkage of *C. albicans* antibodies with schizophrenia, and this association was independent of potential confounders such as age, race, antipsychotic medication, homelessness, socioeconomic status, and a history of cancer. The similarly significant antibody elevation in males with bipolar disorder was found to be purely a function of increased exposures to this pathogen due to a past period of homelessness. In women, we were not able to detect significant differences in strict comparisons of antibody levels among the diagnostic groups. This finding seems logical given that the female reproductive anatomy is prone to yeast infections. Thus, women are likely exposed more often to *C. albicans* overgrowth regardless of psychiatric status and so any disease-related association might be obscured. Intriguingly, however, it was women who exhibited *C. albicans* associations with decreased cognition, suggesting that elimination of the overgrowth might help to improve cognition-associated symptoms. We can speculate that this significant association of yeast antibodies with cognition, which is not observed in control women in spite of an equivalent rate of exposure, is consistent with a gene by environmental etiology of psychiatric disorders.^16,17^ In this scenario, the exposure to an immunopathogenic substance or agent in an individual with a genetically encoded immune system or endothelial barrier defect could be prone to CNS compromise by potentially neurotrophic pathogens or by peripherally generated immune factors.^18,19^ A role for peripherally acting systems in a CNS disorder was also supported by our findings that antibody levels were higher in people who reported GI conditions compared with those who did not. This GI connection was independent of those variables identified as confounders and preliminarily points toward a GI route of *C. albicans* immune response generation, perhaps signaling the presence of gut dysbiosis. Our findings, therefore, may be consistent with the hypothesis that disturbance to the gut microbiome in psychiatric disorders can lead to translocation of gut-related products, including those that are derived from fungi, into systemic circulation.^18–20^

These data also serve in part as biological validation of our recently published next-generation sequencing of the oropharyngeal microbiome, where among several dysregulated bacterial species, we detected elevated abundances of the fungal *Candida spp.*, *C. dubliensis,* in people with schizophrenia compared with controls.^21^ Fungal material has been found in the cerebrospinal fluid and brains of patients with Alzheimer’s disease and amyotrophic lateral sclerosis.^22–24^ Although these recent reports support the possibility that the *C. albicans* organism is directly pathogenic to the brain, yeast alternatively may provide a source of toxic breakdown products or digested bioactive peptides that could elicit an antifungal immune response and have the propensity to cross the blood–gut and blood–brain barriers. Yeasts also synthesize cyclic dipeptides and based on research in other disciplines, it is further possible that these peptides activate apoptotic pathways associated with neuronal function.^25^ Certain metabolic products of yeast including propionic acid have been implicated in autism.^26^ Yeasts are also known producers of neurotransmitters including noradrenaline by *Saccharomyces spp* and serotonin by *Candida spp*.^27^

The hypothesis that infectious agents contribute to the etiopathophysiology of psychiatric disorders is most often applied in a context of pathogen exposure or immune activation during critical prenatal neurodevelopmental time windows.^6,28–35^ It is more difficult to ascertain whether infection or activation of the infectious disease process during one’s lifetime contributes to an increased risk for the development of mental illness. Nevertheless, there is an expansive literature base that aims to understand how pathogen-specific and generalized immune dysregulation extrinsic to the prenatal setting might contribute to future psychiatric disorders.^35–42^ In many of these studies, as well as in our own assays, an IgG antibody-based measure was used and this index does not differentiate current infections from past exposures. In the data not shown, we tested a random sample of 88 individuals for acute *C. albicans* infection using an IgM antibody, a measure that generally reflects a more recent infection. We found that ~9% of these samples were positive for IgM, whereas 32% were positive for IgG, suggesting that for most individuals, *C. albicans* overgrowth occurred sometime in the more distant past. Longitudinal investigations are required to more directly address the issue of postnatal exposures and psychiatric disorder development. Interestingly, studies of military cohorts have documented IgG elevations against a number of antigens, including pathogens, as early as 2 years prior to the diagnosis of disease.^43–45^

Several methodological, analytical, and conceptual factors limit the extent to which our results can be interpreted. From a technical standpoint, given that there are over 150 species of *Candida*, it is possible that the commercial immunoassay kit that we used also detected exposures to other similarly immunogenic Candidal species, including the many that are benign and importantly the few that are pathogenic. In our data analyses, it was not possible for us to correct for all of the multiple variables including lifestyle characteristics that might contribute to our findings. For example, information regarding immunosuppression from sources other than cancer treatment was not available for our study analyses. We addressed potentially confounding basic demographic factors by including these variables in our multiple regressions models. In this manner and as described earlier, we were able to find that a history of homelessness significantly accounted for the elevated *C. albicans* exposures in males with bipolar disorder. Of note, none of the other demographic factors (age, race, and socioeconomic status) were independently or interactively associated with *C. albicans* IgG levels. We applied multiple comparison testing for the basic interdiagnostic group analyses; however, applying this correction to the smaller sample size sub-category comparisons would have eliminated detection of informative associations. Therefore, such sub-analyses including the GI associations remain preliminary and support the exploratory nature of this paper. Although our results demonstrate an absence of effect of antipsychotic medications on *C. albicans* IgG levels, we did not have information regarding over-the-counter or prescription treatments of the somatic conditions evaluated in this study.

In conclusion, our initial results suggested the absence of differential *C. albicans* IgG levels between psychiatric cases and controls. However, when sexes were evaluated separately, numerous significant disease-specific associations of this fungus with lifestyle, somatic conditions, and cognitive measures were evident. It may be premature to list this pathogen as a risk factor for disease causation, but its status as a comorbidity requires clinical attention. During health care evaluation, inquiry regarding the presence of symptoms of *C. albicans* infections would help to identify individuals in need of treatment. Furthermore, the early identification of active infection might be an indicator of those at risk for cognitive decline; however, future research is necessary to investigate this connection. In people who have or who are prone to *C. albicans* overgrowth, dietary management geared toward correcting microbial imbalances may be considered. In the long term, more research is required to understand the mechanisms that trigger pathogenicity of fungal commensals and how this might impact brain function in psychiatric disorders.

## Materials and Methods

### Study participants

#### Cohort 1—Sheppard Pratt Health System, Baltimore, MD, USA

This study cohort was composed of 808 individuals: 277 were control individuals with no history of psychiatric disorder; 261 individuals were diagnosed with schizophrenia; and 270 were diagnosed with bipolar disorder. Diagnoses were made according to criteria defined by DSM-IV-TR^46^ and have been previously described.^47,48^ For inclusion in the schizophrenia group, individuals received a DSM-IV-TR diagnosis of schizophrenia, schizophreniform disorder, or schizoaffective disorder. Individuals with bipolar disorder had a DSM-IV-TR diagnosis of type 1 or type 2 bipolar disorder or bipolar disorder not otherwise specified. For both groups, inclusion criteria required an age between 18 and 65 years. Individuals without a history of psychiatric disorder were recruited from posted announcements and were screened to rule out current or past psychiatric disorders with the Structured Clinical Interview for DSM-IV Axis I Disorders Non-Patient Edition.^49^ Control participants were between the ages of 20 and 60 years, inclusive. Exclusion criteria for all groups included: mental retardation; clinically significant medical disorder that would affect cognitive performance; any history of intravenous substance abuse or a primary diagnosis of substance abuse or substance dependence. For controls, any active substance misuse was considered an exclusion criterion.

At the time of interview, cognitive functioning was assessed with the RBANS Form A.^15^ Clinical information was gathered, and individuals were asked to report current or chronic conditions related to their health. These conditions could include those that occurred within the last month, were treated with medication, or were chronic. Medical records and self-reports were reviewed for the following categories: cardiovascular, endocrine, genitourinary, GI, hepatological, neoplastic, and respiratory disturbances. Specific conditions falling within each category are reported in the Results section for those categories that exhibited significant case–control differences. Information for these categories was not available for all individuals, but relevant sample sizes are reported in the Results section. Basic demographic data for this study population are shown in Table 4. Maternal education was used as a surrogate for socioeconomic status. Diagnostic groups differed significantly in age, homelessness, race, sex and maternal education. Thus, these variables were all included in the multivariate analyses described below.

These studies were approved by the Institutional Review Boards of the Sheppard Pratt Health System and the Johns Hopkins Medical Institution following established guidelines. All participants provided written informed consent after study procedures were explained. This research was performed in accordance with The Code of Ethics of the World Medical Association (Declaration of Helsinki) for experiments involving humans.

#### Cohort 2—University of Cologne, Cologne, Germany

The methods for identifying and characterizing the individuals with a first episode of schizophrenia according to criteria defined by DSM-IV have also been previously described.^50^ Seventy-eight of these patients were antipsychotic naive and 61 patients were currently receiving antipsychotic medication. Individuals were excluded from the study if they had a relevant comorbidity such as heart disease, liver cirrhosis, known immune-mediated disease (such as multiple sclerosis), or a history of substance dependence. Demographic data regarding age and sex are listed in Table 4. The region from which patients were recruited was generally homogenous regarding socioeconomic characteristics. Informed consent was obtained from all study participants. Protocols for sample collection and analyses were approved by the ethics committee at the University of Cologne, Heidelberg University and Johns Hopkins University, in accordance with the Declaration of Helsinki.

### Laboratory procedures

Blood samples were obtained by venipuncture, and plasma and serum separated and assessed for antibodies. Anti-*C. albicans* IgG levels were measured according to the manufacturer’s protocol using a commercially available kit (Abcam, Cambridge, MA, USA). Each 96-well plate tested contains kit standards as well as study sample replicates for use as internal controls of reproducibility.

### Statistical analyses

Following test procedures, investigators were unblinded for the data analyses. Differences in quantitative levels of antibodies between diagnostic groups were compared using ANOVAs with Sidak *post hoc* tests. *C. albicans* IgG seropositivity was designated using three different cutoff values to best capture a range of positivities: (1) absorbance value below which 90% of female control IgG values fall; (2) absorbance value below which 90% of male control IgG values fall; and (3) absorbance value designations assigned by standards of the commercial ELISA kit included for each plate. Higher absorbance values utilized for a cutoff are more conservative than the lower absorbance values that are less rigorous estimates of seropositivity (Figure 1). Odds ratios for disease association of IgG quantitative levels and the three differently calculated IgG seropositivities were assigned using multivariate logistic regressions corrected for age, homelessness, sex, race, and maternal education. Paired comparisons between groups of other continuous variables including age and RBANS scores were also evaluated with *t*-tests. Multiple comparison testing was evaluated and applied to each analysis depending on the number of comparisons. Results that were significant but fell short of Bonferroni *P*-value limits are so indicated. Differences in categorical demographic data were evaluated with chi square tests. All the data analyses were performed with groups subdivided according to sex so that sex-specific differences could be identified. For multivariate regressions, sex was dropped as a correction variable when all members of the same sex were evaluated. Statistical analyses were performed with STATA version 12 (STATA Corp LP, College Station, TX, USA).

### Availability of the data and materials

Data generated from this study will be made available upon request. Samples may be made available upon request provided that conditions relating to Institutional Review Boards and MTAs at cooperating institutions are met.

## Figures and Tables

**Table 1:** *C. albicans* positivity confers increased odds for schizophrenia in males

*Sex*	*Diagnostic group*	*C. albicans Positivity Model*^a^	*Odds ratio*	*95% confidence interval*	P*-value*
Female	SCH	a	1.39	0.58–3.35	0.47
		b	1.26	0.67–2.37	0.47
		c	1.25	0.70–2.21	0.45
		d	1.12	0.66–1.90	0.67
Female	BPD	a	0.59	0.25–1.41	0.24
		b	0.81	0.49–1.33	0.40
		c	0.88	0.58–1.36	0.58
		d	0.74	0.47–1.17	0.20
Male	SCH	a	9.53	1.19–76.50	**0.0001**
		b	2.88	1.36–6.13	**0.0001**
		c	2.04	1.19–3.51	**0.0001**
		d	3.32	1.55–7.11	**0.0001**
Male	BPD	a	2.45	0.23–25.99	0.46
		b	2.00	0.82–4.88	0.13
		c	1.47	0.76–2.86	0.26
		d	1.73	0.68–4.36	0.25

Abbreviations: BPD, bipolar disorder; SCH, schizophrenia.

a As described in the Materials and Methods section, positivity models are based on dichotomous 90% control female values (a), dichotomous 90% male control values (b), dichotomous cutoff values designated by standards contained in the commercial ELISA kit (c), and continuous quantitative antibody levels (d). Multinomial logistic regressions of diagnostic group with *C. albicans* positivity were corrected for age, gender, homelessness and socioeconomic status. Female and male non-psychiatric controls are the respective comparison groups. Statistically significant *P*-values are bolded.

**Table 2:** *C. albicans* positivity-associated memory deficits in females with schizophrenia and bipolar disorder

*RBANS module*	*Diagnostic group*	*C. albicans Positivity Model*^a^	*C. albicans negative*			*C. albicans positive*			*t*	*One-tail* P*-value*	*Two-tail* P*-value*
			n	*Mean*	*s.d.*	n	*Mean*	*s.d.*			
Immediate memory	CON	a	155	92.45	13.9	14	96.14	13.93	−0.95	0.17	0.34
		b	124	92.06	14.13	45	94.67	13.21	−1.08	0.14	0.28
		c	79	93.30	14.28	90	92.27	13.62	0.48	0.31	0.63
	SCH	a	87	68.54	16.98	13	57.38	10.93	2.29	**0.01**	**0.02**
		b	73	67.11	16.19	27	67.04	18.36	0.02	0.49	0.98
		c	46	68.02	16.73	54	66.30	16.81	0.51	0.31	0.61
	BPD	a	182	83.48	16.08	11	86.00	13.83	−0.51	0.31	0.61
		b	148	84.78	15.79	45	79.80	15.98	1.85	**0.033**	0.07
		c	96	86.68	14.52	97	80.60	16.76	2.69	**0.004**	**0.008**
Delayed memory	CON	a	155	89.65	8.99	14	93.64	6.65	−1.62	0.053	0.11
		b	124	89.57	9.14	45	91.09	8.09	−0.98	0.16	0.33
		c	79	90.35	7.90	90	89.64	9.68	0.52	0.30	0.61
	SCH	a	87	71.39	15.50	13	56.23	16.03	3.27	**0.0007**	**0.001**
		b	73	70.74	16.39	27	65.85	15.84	1.34	0.09	0.18
		c	46	71.39	17.15	54	67.74	15.52	1.12	0.13	0.27
	BPD	a	182	81.67	15.66	11	80.64	19.03	0.21	0.42	0.83
		b	148	82.51	15.30	45	78.64	17.25	1.44	0.08	0.15
		c	96	84.09	14.39	97	79.15	16.83	2.19	**0.01**	**0.03**

Abbreviations: BPD, bipolar disorder; CON, non-psychiatric control; SCH, schizophrenia.

a As described in the Materials and Methods section, positivity models are based on dichotomous 90% control female values (a), dichotomous 90% male control values (b), and dichotomous cutoff values designated by standards contained in the commercial ELISA kit (c). Total RBANS scores were lower in females with schizophrenia compared with controls (see main text). Statistically significant *P*-values are bolded.

**Table 3:** Somatic conditions associated with *C. albicans* IgG levels in schizophrenia and bipolar disorder

*Somatic condition*	*Sex*	*Diagnostic group*	*Condition—negative*			*Condition—positive*			*t*	*One-tail* P*-value*	*Two-tail* P*-value*
			n	*Mean C. albicans IgG*	*s.d.*	n	*Mean C. albicans IgG*	*s.d.*			
GI	Female	SCH	38	0.62	0.40	62	0.72	0.61	−0.92	0.18	0.36
GI	Female	BPD	80	0.52	0.39	113	0.68	0.48	−2.41	**0.009**	**0.02**
GI	Male	SCH	82	0.53	0.41	79	0.70	0.48	−2.42	**0.008**	**0.02**
GI	Male	BPD	39	0.55	0.43	38	0.66	0.48	−1.1	0.14	0.27
G-UR	Female	SCH	55	0.70	0.58	45	0.66	0.49	0.39	0.35	0.70
G-UR	Female	BPD	90	0.55	0.45	101	0.68	0.45	−1.95	**0.03**	**0.05**
G-UR	Male	SCH	122	0.58	0.42	39	0.72	0.52	−1.75	**0.04**	0.08
G-UR	Male	BPD	60	0.61	0.47	17	0.59	0.42	0.17	0.43	0.86
NEO	Female	SCH	93	0.69	0.54	7	0.54	0.48	0.71	0.24	0.48
NEO	Female	BPD	164	0.61	0.44	28	0.64	0.54	−0.37	0.36	0.71
NEO	Male	SCH	155	0.60	0.45	5	0.91	0.56	−1.53	**0.06**	0.13
NEO	Male	BPD	71	0.59	0.45	6	0.77	0.48	−0.93	0.18	0.35

Abbreviations: BPD, bipolar disorder; GI, gastrointestinal; G-UR, genitourinary; NEO, neoplastic; SCH, schizophrenia. Statistically significant *P*-values are bolded.

**Table 4:** Demographics of the study populations

*Population*	n	*Age Mean years*	*Age s.d.*	*Gender n (% Female)*	*Homeless n (% with history)*	*Race n (% Caucasian)*	*Maternal education Mean years*	*Maternal education s.d.*
*Cohort 1*								
CON^a^	277	32.02	11.31	169 (61.0)	2 (0.7)	138 (49.8)	13.70	2.68
SCH	261	37.71^b^	13.69	100 (38.3)^c^	52 (19.9)^d^	125 (47.9)	12.85^e^	2.66
BPD	270	34.08^f^	13.15	193 (71.5)^g^	26 (9.6)^h^	187 (69.3)^i^	13.36	3.16
								
*Cohort 2*								
FEP SCH AP-	78	30.34^j^	10.60	34 (43.6)	NA	NA	NA	NA
FEP SCH AP+	61	36.49	13.17	31 (50.8)	NA	NA	NA	NA

Abbreviations: AP−, antipsychotic naive; AP+, antipsychotic positive; BPD, bipolar disorder; CON, non-psychiatric control; FEP, first episode; SCH, schizophrenia.

a Cohort 1 tests are SCH and BPD compared with controls. Cohort 2 tests are AP− compared with AP+.

b *t*=5.26, two-tail *P*⩽0.0001.

c *χ* ^2^=27.69, *P*⩽0.001.

d *χ* ^2^=54.87, *P*⩽0.001.

e *t*=3.70, two-tail *P*⩽0.0002.

f *t*=1.96, two-tail *P*⩽0.05.

g *χ* ^2^= 6.70, *P*⩽0.01.

h *χ* ^2^= 22.34, *P*⩽0.001.

i *χ* ^2^=21.43, *P*⩽0.001.

j *t*=3.04, two-tail *P*⩽0.003.

## References

[bib1] Horvath, S. & Mirnics, K. Immune system disturbances in schizophrenia. Biol. Psychiatry 75, 316–323 (2014). 23890736 10.1016/j.biopsych.2013.06.010 PMC3841236

[bib2] Muller, N. Immunology of schizophrenia. Neuroimmunomodulation 21, 109–116 (2014). 24557043 10.1159/000356538

[bib3] Consortium SWGotPG. Biological insights from 108 schizophrenia-associated genetic loci. Nature 511, 421–427 (2014). 25056061 10.1038/nature13595 PMC4112379

[bib4] Arias, I. et al. Infectious agents associated with schizophrenia: a meta-analysis. Schizophr. Res. 136, 128–136 (2012). 22104141 10.1016/j.schres.2011.10.026

[bib5] Yolken, R. H. & Torrey, E. F. Are some cases of psychosis caused by microbial agents? A review of the evidence. Mol. Psychiatry 13, 470–479 (2008). 18268502 10.1038/mp.2008.5

[bib6] Knuesel, I. et al. Maternal immune activation and abnormal brain development across CNS disorders. Nat. Rev. Neurol. 10, 643–660 (2014). 25311587 10.1038/nrneurol.2014.187

[bib7] Labouesse, M. A. , Langhans, W. & Meyer, U. Long-term pathological consequences of prenatal infection: beyond brain disorders. Am. J. Physiol. Regul. Integr. Comp. Physiol. 309, R1–R12 (2015). 25924881 10.1152/ajpregu.00087.2015

[bib8] Severance, E. G. et al. Gastrointestinal inflammation and associated immune activation in schizophrenia. Schizophr. Res. 138, 48–53 (2012). 22446142 10.1016/j.schres.2012.02.025 PMC4244845

[bib9] Severance, E. G. et al. Seroreactive marker for inflammatory bowel disease and associations with antibodies to dietary proteins in bipolar disorder. Bipolar Disord. 16, 230–240 (2014). 24313887 10.1111/bdi.12159 PMC4075657

[bib10] Ghannoum, M. A. et al. Characterization of the oral fungal microbiome (mycobiome) in healthy individuals. PLoS Pathog. 6, e1000713 (2010). 20072605 10.1371/journal.ppat.1000713 PMC2795202

[bib11] Suhr, M. J. & Hallen-Adams, H. E. The human gut mycobiome: pitfalls and potentials-a mycologist's perspective. Mycologia 107, 1057–1073 (2015). 26354806 10.3852/15-147

[bib12] Kim, J. & Sudbery, P. *Candida albicans*, a major human fungal pathogen. J. Microbiol. 49, 171–177 (2011). 21538235 10.1007/s12275-011-1064-7

[bib13] Mukherjee, P. K. et al. Mycobiota in gastrointestinal diseases. Nat. Rev. Gastroenterol. Hepatol. 12, 77–87 (2015). 25385227 10.1038/nrgastro.2014.188

[bib14] Murphy, K. et al. Janeway's Immunobiology, Eighth edition (Garland Science, 2012).

[bib15] Randolph, C. RBANS Manual—Repeatable Battery for the Assessment of Neuropsychological Status. (Psychological Corporation, 1998).

[bib16] Demjaha, A. , MacCabe, J. H. & Murray, R. M. How genes and environmental factors determine the different neurodevelopmental trajectories of schizophrenia and bipolar disorder. Schizophr. Bull. 38, 209–214 (2012). 21857009 10.1093/schbul/sbr100 PMC3283142

[bib17] van Os, J. et al. Identifying gene-environment interactions in schizophrenia: contemporary challenges for integrated, large-scale investigations. Schizophr. Bull. 40, 729–736 (2014). 24860087 10.1093/schbul/sbu069 PMC4059449

[bib18] Severance, E. G. , Prandovszky, E. , Castiglione, J. & Yolken, R. H. Gastroenterology issues in schizophrenia: why the gut matters. Curr. Psychiatry Rep. 17, 27 (2015). 25773227 10.1007/s11920-015-0574-0 PMC4437570

[bib19] Severance E. G. , Yolken R. H . & Eaton W. W . Autoimmune diseases, gastrointestinal disorders and the microbiome in schizophrenia: more than a gut feeling. Schizophr. Res. (2014) (e-pub ahead of print). 10.1016/j.schres.2014.06.027 PMC4294997 25034760

[bib20] Severance, E. G. et al. Discordant patterns of bacterial translocation markers and implications for innate immune imbalances in schizophrenia. Schizophr. Res. 148, 130–137 (2013). 23746484 10.1016/j.schres.2013.05.018 PMC3732507

[bib21] Castro-Nallar, E. et al. Composition, taxonomy and functional diversity of the oropharynx microbiome in individuals with schizophrenia and controls. PeerJ 3, e1140 (2015). 26336637 10.7717/peerj.1140 PMC4556144

[bib22] Pisa, D. , Alonso, R. , Rabano, A. , Rodal, I. & Carrasco, L. Different brain regions are infected with fungi in Alzheimer's disease. Sci. Rep. 5, 15015 (2015). 26468932 10.1038/srep15015 PMC4606562

[bib23] Alonso, R. , Pisa, D. , Rabano, A. , Rodal, I. & Carrasco, L. Cerebrospinal fluid from Alzheimer's Disease patients contains fungal proteins and DNA. J. Alzheimers Dis. 47, 873–876 (2015). 26401766 10.3233/JAD-150382

[bib24] Alonso, R. et al. Evidence for fungal infection in cerebrospinal fluid and brain tissue from patients with amyotrophic lateral sclerosis. Int. J. Biol. Sci. 11, 546–558 (2015). 25892962 10.7150/ijbs.11084 PMC4400386

[bib25] Semon, B. A. Dietary cyclic dipeptides, apoptosis and psychiatric disorders: a hypothesis. Med. Hypotheses 82, 740–743 (2014). 24717821 10.1016/j.mehy.2014.03.016

[bib26] Burrus, C. J. A biochemical rationale for the interaction between gastrointestinal yeast and autism. Med. Hypotheses 79, 784–785 (2012). 23021572 10.1016/j.mehy.2012.08.029

[bib27] Dinan, T. G. , Borre, Y. E. & Cryan, J. F. Genomics of schizophrenia: time to consider the gut microbiome? Mol. Psychiatry 19, 1252–1257 (2014). 25288135 10.1038/mp.2014.93

[bib28] Buka, S. L. , Cannon, T. D. , Torrey, E. F. & Yolken, R. H. Maternal exposure to herpes simplex virus and risk of psychosis among adult offspring. Biol. Psychiatry 63, 809–815 (2008). 17981263 10.1016/j.biopsych.2007.09.022

[bib29] Buka, S. L. et al. Maternal cytokine levels during pregnancy and adult psychosis. Brain Behav. Immun. 15, 411–420 (2001). 11782107 10.1006/brbi.2001.0644

[bib30] Severance, E. G. , Gressitt, K. L. , Buka, S. L. , Cannon, T. D. & Yolken, R. H. Maternal complement C1q and increased odds for psychosis in adult offspring. Schizophr. Res. 159, 14–19 (2014). 25195065 10.1016/j.schres.2014.07.053 PMC4177507

[bib31] Brown, A. S. et al. Serologic evidence of prenatal influenza in the etiology of schizophrenia. Arch. Gen. Psychiatry 61, 774–780 (2004). 15289276 10.1001/archpsyc.61.8.774

[bib32] Brown, A. S. & Derkits, E. J. Prenatal infection and schizophrenia: a review of epidemiologic and translational studies. Am. J. Psychiatry 167, 261–280 (2010). 20123911 10.1176/appi.ajp.2009.09030361 PMC3652286

[bib33] Brown, A. S. & Susser, E. S. In utero infection and adult schizophrenia. Ment. Retard. Dev. Disabil. Res. Rev. 8, 51–57 (2002). 11921387 10.1002/mrdd.10004

[bib34] Canetta, S. E. & Brown, A. S. Prenatal infection, maternal immune activation, and risk for schizophrenia. Transl. Neurosci. 3, 320–327 (2012). 23956839 10.2478/s13380-012-0045-6 PMC3744366

[bib35] Blomstrom, A. et al. Associations between maternal infection during pregnancy, childhood infections, and the risk of subsequent psychotic disorder-A Swedish Cohort Study of nearly 2 million individuals. Schizophr. Bull. 42, 125–133 (2016). 26303935 10.1093/schbul/sbv112 PMC4681563

[bib36] Markovitz, A. A. et al. *Toxoplasma gondii* and anxiety disorders in a community-based sample. Brain Behav. Immun. 43, 192–197 (2015). 25124709 10.1016/j.bbi.2014.08.001

[bib37] Khandaker, G. M. , Stochl, J. , Zammit, S. , Lewis, G. & Jones, P. B. Childhood Epstein-Barr Virus infection and subsequent risk of psychotic experiences in adolescence: a population-based prospective serological study. Schizophr. Res. 158, 19–24 (2014). 25048425 10.1016/j.schres.2014.05.019 PMC4561501

[bib38] Benros, M. E. et al. Autoimmune diseases and severe infections as risk factors for schizophrenia: a 30-year population-based register study. Am. J. Psychiatry 168, 1303–1310 (2011). 22193673 10.1176/appi.ajp.2011.11030516

[bib39] Dalman, C. et al. Infections in the CNS during childhood and the risk of subsequent psychotic illness: a cohort study of more than one million Swedish subjects. Am. J. Psychiatry 165, 59–65 (2008). 18056223 10.1176/appi.ajp.2007.07050740

[bib40] Nielsen, P. R. , Benros, M. E. & Mortensen, P. B. Hospital contacts with infection and risk of schizophrenia: a population-based cohort study with linkage of Danish national registers. Schizophr. Bull. 40, 1526–1532 (2014). 24379444 10.1093/schbul/sbt200 PMC4193697

[bib41] Blomstrom, A. et al. Hospital admission with infection during childhood and risk for psychotic illness--a population-based cohort study. Schizophr. Bull. 40, 1518–1525 (2014). 24366719 10.1093/schbul/sbt195 PMC4193695

[bib42] Khandaker, G. M. , Zimbron, J. , Dalman, C. , Lewis, G. & Jones, P. B. Childhood infection and adult schizophrenia: a meta-analysis of population-based studies. Schizophr. Res. 139, 161–168 (2012). 22704639 10.1016/j.schres.2012.05.023 PMC3485564

[bib43] Li, Y. et al. Association between antibodies to multiple infectious and food antigens and new onset schizophrenia among US military personnel. Schizophr. Res. 151, 36–42 (2013). 24139899 10.1016/j.schres.2013.10.004

[bib44] Niebuhr, D. W. et al. Selected infectious agents and risk of schizophrenia among U.S. military personnel. Am. J. Psychiatry 165, 99–106 (2008). 18086751 10.1176/appi.ajp.2007.06081254

[bib45] Niebuhr, D. W. , Millikan, A. M. , Yolken, R. , Li, Y. & Weber, N. S. Results from a hypothesis generating case-control study: herpes family viruses and schizophrenia among military personnel. Schizophr. Bull. 34, 1182–1188 (2008). 18156638 10.1093/schbul/sbm139 PMC2632504

[bib46] APA. Diagnostic and Statistical Manual of Mental Disorders: DSM-IV-TR, 4th edn (American Psychiatric Association, 2000).

[bib47] Dickerson, F. et al. Pentraxin 3 is reduced in bipolar disorder. Bipolar Disord. 17, 409–414 (2015). 25425421 10.1111/bdi.12281

[bib48] Dickerson, F. et al. C-reactive protein is elevated in schizophrenia. Schizophr. Res. 143, 198–202 (2013). 23218564 10.1016/j.schres.2012.10.041

[bib49] First, M. B. , Spitzer, R. L. , Gibbon, M. & Williams, J. B. W. Structured Clinical Interview for DSM-IV Axis I Disorders—Non-patient Edition (SCID I/NP). (Biometrics Research, New York State Psychiatric Institute, 1998).

[bib50] Leweke, F. M. et al. Antibodies to infectious agents in individuals with recent onset schizophrenia. Eur. Arch. Psychiatry Clin. Neurosci. 254, 4–8 (2004). 14991372 10.1007/s00406-004-0481-6

